# Isolation and characterization of new *Puumala orthohantavirus* strains from Germany

**DOI:** 10.1007/s11262-020-01755-3

**Published:** 2020-04-23

**Authors:** Florian Binder, Sven Reiche, Gleyder Roman-Sosa, Marion Saathoff, René Ryll, Jakob Trimpert, Dusan Kunec, Dirk Höper, Rainer G. Ulrich

**Affiliations:** 1 Institute of Novel and Emerging Infectious Diseases, Federal Research Institute for Animal Health, Friedrich-Loeffler-Institut, Greifswald - Insel Riems, Germany; 2 Department of Experimental Animal Facilities and Biorisk Management, Federal Research Institute for Animal Health, Friedrich-Loeffler-Institut, Greifswald - Insel Riems, Germany; 3 Institute of Diagnostic Virology, Federal Research Institute for Animal Health, Friedrich-Loeffler-Institut, Greifswald - Insel Riems, Germany; 4 Department of Diagnostic Medicine/Pathobiology, Kansas State University, Manhattan, KS USA; 5 Specialist Pest Control, Veterinary Task Force, Lower Saxony State Office for Consumer Protection and Food Safety, Oldenburg, Germany; 6 Department of Veterinary Medicine, Institute of Virology, Freie Universität Berlin, Berlin, Germany; 7 German Center for Infection Research (DZIF), Partner Site Hamburg-Lübeck-Borstel-Insel Riems, Greifswald - Insel Riems, Germany

**Keywords:** *Puumala orthohantavirus*, Bank vole, Cell culture, Virus adaptation, Glycoprotein-specific antibodies

## Abstract

Orthohantaviruses are re-emerging rodent-borne pathogens distributed all over the world. Here, we report the isolation of a *Puumala orthohantavirus* (PUUV) strain from bank voles caught in a highly endemic region around the city Osnabrück, north-west Germany. Coding and non-coding sequences of all three segments (S, M, and L) were determined from original lung tissue, after isolation and after additional passaging in VeroE6 cells and a bank vole-derived kidney cell line. Different single amino acid substitutions were observed in the RNA-dependent RNA polymerase (RdRP) of the two stable PUUV isolates. The PUUV strain from VeroE6 cells showed a lower titer when propagated on bank vole cells compared to VeroE6 cells. Additionally, glycoprotein precursor (GPC)-derived virus-like particles of a German PUUV sequence allowed the generation of monoclonal antibodies that allowed the reliable detection of the isolated PUUV strain in the immunofluorescence assay. In conclusion, this is the first isolation of a PUUV strain from Central Europe and the generation of glycoprotein-specific monoclonal antibodies for this PUUV isolate. The obtained virus isolate and GPC-specific antibodies are instrumental tools for future reservoir host studies.

**Electronic supplementary material:**

The online version of this article (10.1007/s11262-020-01755-3) contains supplementary material, which is available to authorized users.

## Introduction

*Puumala orthohantavirus* (PUUV) is the most important hantavirus in Europe [1]. It causes the majority of human hantavirus infections and hemorrhagic fever with renal syndrome (HFRS) cases [2]. In Central and Western Europe hantavirus outbreaks occur in two to five year intervals and are driven by massive increase of the bank vole (*Myodes glareolus*) population, the reservoir of this orthohantavirus species [3]. Human hantavirus disease is notifiable in Germany since 2001 and the majority of recorded cases is mainly due to PUUV infections in southern and western parts of Germany, whereas *Dobrava-Belgrade orthohantavirus* (DOBV) with the striped field mouse as reservoir causes infections in the northeastern part of Germany [3].

The characterization of the pathogenicity and identification of virulence markers are highly dependent on adequate PUUV isolates. Currently, the number of PUUV isolates is very limited and does not represent the real diversity of PUUV strains in Europe. In particular, no Central European PUUV isolate exists [4]. The majority of PUUV isolates, and hantaviruses in general, was obtained based on passaging in reservoir animals or VeroE6 cells and is highly adapted [5–7]. Previous investigations indicated that VeroE6 cell adaptation of PUUV Kazan strain results in the inability of the adapted strain to infect the bank vole reservoir [8]. The recent development of bank vole-derived primary or permanent cell lines may allow the isolation of reservoir-adapted PUUV strains [9–12].

Hantavirus proteins are usually detected in infected cells by monoclonal antibodies. Nucleocapsid (N) protein-specific monoclonal antibodies have been developed against a large range of hantaviruses [13–15]. In contrast, the number of glycoprotein precursor (GPC), as well as Gc- and Gn-specific monoclonal antibodies is rather low [16–18]. The majority of these antibodies were raised by infection of bank voles or immunization with recombinant N protein or heterologous virus-like particles (VLPs). The generation of envelope protein-specific monoclonal antibodies with reactivity to virus proteins in infected cells is highly dependent on structural constraints [19]. Autologous VLPs represent a useful tool to generate highly efficient immune responses against a variety of viruses and for the generation of monoclonal antibodies in particular [20]. PUUV strain Astrup [21] GPC-derived VLPs were generated in this study as previously described for Maporal orthohantavirus [22].

Lower Saxony, north-west Germany, and district Osnabrück in particular, is a well-known endemic region for PUUV infections [23, 24]. This endemic region was also again heavily affected by the hantavirus outbreak year 2019 [25]. Here, we aimed to isolate a Central European PUUV strain from bank voles in the district of Osnabrück using standard VeroE6 cells and the recently established Carpathian lineage bank vole-derived kidney cell line (MGN-2-R [10]). Complete genome determination by shot-gun and hybrid-capture-mediated high-throughput sequencing (HTS) was used to follow the potential adaptation of the PUUV isolates in VeroE6 and reservoir cell lines. Finally, the reactivity of the isolates was determined with novel monoclonal antibodies raised against PUUV GPC VLPs.

## Materials and methods

### Trapping and dissection

Bank voles were trapped in spring 2019 in the PUUV endemic region around Osnabrück following a standard snap trapping protocol [25, 26]. In the field, a small piece of lung was taken for virus isolation and RT-qPCR analysis. Thereafter, carcasses were frozen, transported to the laboratory and completely dissected according to standard protocols. Chest cavity lavage was collected by rinsing the chest cavity by 1 ml phosphate-buffered saline (PBS) and investigated for the presence of PUUV-reactive antibodies. The presence of hantavirus RNA was analyzed from lung tissue and were, in part, previously published in a surveillance study [25].

### Cell lines

For virus isolation and further infection studies, VeroE6 and bank vole kidney (MGN-2-R; [10]) cells were used in parallel. Virus titration was done on VeroE6 cells only. MGN-2-R cells were grown in an equal mixture of Hams’ F12 and Iscove’s modified Dulbecco’s medium (IMDM) + 10% fetal calf serum (FCS) and passaged two times per week at a 1:6 ratio. VeroE6 cells were passaged twice a week in minimal essential medium (MEM) + 10% fetal calf serum (FCS) and a split ratio of 1:4.

### Virus isolation

For virus isolation, 1 × 10^5^ MGN-2-R or VeroE6 cells were seeded in 12.5 cm^2^ flasks one day before rodent sampling in the field. The cells were carried to trapping sites in an isolation box with heat packs (around 33 °C constant for 2 days with outside temperature of 5–10 °C). After collecting voles from traps, a small incision in the chest area was made and a piece of lung (pea-sized) was taken and transferred into 1 ml Dulbecco's Modified Eagle's Medium (DMEM) + 5% FCS + penicillin/streptomycin (PS) in a 5 ml safe lock tube. Lung tissue material was homogenized in the field by grinding it through a fine metal grid against the tube wall. The homogenized tissue material was sterile filtered (0.45 µm) directly onto the cells resulting in approximately 500 µl tissue/medium suspension per 12.5 cm^2^ flask. After 1–2 h incubation in the isolation box, 4 ml DMEM + 5% FCS + PS was added. Upon arrival in the laboratory flasks were incubated in a cell culture incubator at 37 °C and 5% CO_2_ for 10 days until first passage. In parallel, a pinhead-sized piece of lung was taken for RNA isolation in 1 ml Trizol (QIAGEN, Hilden, Germany).

After 10 days, trypsinized cells were resuspended in 2 ml DMEM + 5% FCS + PS. For PUUV RNA screening, 325 µl of each cell suspension was taken for RNA extraction and analyzed by RT-qPCR (see below). Fresh VeroE6 cells were resuspended in 2 ml DMEM + 5% FCS + PS and 200 µl were mixed 1:1 with 200 µl of the inoculated cell suspension in a new 12.5 cm^2^ flask. Afterwards, 4 ml DMEM + 5% FCS + PS were added and cells were incubated for 10 days until next passage. In parallel, one uninfected flask of VeroE6 or MGN-2-R cells was passaged as a control. This procedure was continued until RT-qPCR-positive samples were detected. After first screening, only the flasks of the RT-qPCR-positive samples were further passaged.

### Hantavirus RNA detection

For detection of PUUV nucleic acid, RNA was extracted from homogenized lung tissue, or cell culture passages using QIAzol Lysis Reagent (QIAGEN, Hilden, Germany) followed by a novel PUUV S segment-specific RT-qPCR. For RT-qPCR, primers PUUV-NSs-s (5′-GWNATARCYCGYCATGARC-3′) and PUUV-NSs-as (5′-ARTGCTGACACTGTYTGTTG-3′) and the probe (5′-6-FAM-CRGTGGRRRTGKACCCRGATGA-BHQ-1-3′) were used. The PCR was done according to the QuantiTect Probe One-Step RT-qPCR Mix (QIAGEN, Hilden Germany) protocol and contained 20 pmol/µl of each primer and 5 pmol/µl probe (Eurofins, Hamburg, Germany). The following cycler protocol was used: 30 min of reverse transcription at 50 °C; 15 min initial denaturation at 95 °C; 45 cycles of 10 sec at 95 °C, 25 sec at 50 °C and 25 sec at 72 °C. For quantification of the number of RNA copies/µl and sample, an in vitro transcribed RNA was used. The in vitro transcription of a plasmid coding for nucleotides 83–355 of the S segment of a PUUV strain from Baden-Wuerttemberg (Binder et al., unpublished) was done according to the protocol of the manufacturer (Riboprobe® in vitro Transcription System T7, Promega GmbH, Mann-heim, Germany). The transcribed RNA was serially diluted from 10^–2^ to 10^–11^ ng/ml with 700 RNA copies/µl limit of detection (LOD). Initial tissue samples were screened for PUUV RNA and viral load as RNA copies/µl was determined in triplicates for organs of isolated positive animals. RNA from the cell culture adapted strains PUUV Sotkamo and TULV Moravia were used as positive and negative control for the RT-qPCR, respectively.

### Library preparation, target enrichment, sequencing and analysis

For metagenomics, we extracted RNA from either a pinhead-sized piece of lung tissue or 250 µl cell culture supernatant using 750 µl QIAzol Lysis Reagent (QIAGEN, Hilden, Germany) in combination with RNeasy Mini Kit (QIAGEN, Hilden, Germany). For generation of complete genomes of cell culture supernatants, a previously published workflow was used [27]. Double-stranded, non-directional cDNA libraries from lung tissue for sequencing on the Illumina platform were prepared from total RNA using the NEBNext Ultra II RNA Library Prep Kit for Illumina (New England Biolabs, Ipswich, MA, USA). Per reaction, a total of 100 ng RNA was used as an input. RNA was fragmented for 8 min and final cDNA libraries were amplified by 8 cycles of PCR to complete adapter ligation and to generate enough material for target sequence enrichment. A custom-made myBaits target capture array (Arbor Biosciences, Ann Arbor, MI, USA), containing biotinylated RNA probes against all available PUUV sequences deposited in NCBI GenBank database (August, 2018), was employed to capture PUUV-containing sequences from total cellular cDNA sequencing libraries. The hybridization-based sequence enrichment (chemistry v3) was performed according to the manufacturer’s instructions (Arbor Biosciences, Ann Arbor, MI, USA). The enriched cDNA sequencing libraries were amplified with 14 PCR cycles to produce enough DNA material for HTS on the Illumina platform. The enriched cDNA libraries were quantified with the NEBNext Library Quantification Kit (New England Biolabs, Ipswich, MA, USA), pooled in equimolar amounts, and sequenced with a 600 cycle MiSeq Reagent Kit v3 (Illumina, San Diego, CA, USA) using paired-end sequencing (2 × 300 cycles) on a MiSeq sequencer (Illumina, San Diego, CA, USA). The resulting reads were trimmed and assembled against the known complete genome of strain Astrup from the Osnabrück region [21] with Geneious R11.1.5 (https://www.geneious.com). For sequences lacking the 5′ and 3′ ends of the M segment, RNA ligation was done using T4 RNA Ligase (Thermo Fisher Scientific, Waltham, MA, USA) and subsequent in vitro transcription with a First Strand cDNA Synthesis Kit (Thermo Fisher Scientific, Waltham, MA, USA). Sequences were obtained by conventional dideoxy-chain termination sequencing after PCR with primers PUUV OS M2 fwd-5′ TGAGGGCAATTATTATGTAA 3′ and PUUV OS M2 rev 5′ CCAATTGTATGTGGGCATTCC 3′. The obtained sequences were deposited at GenBank, accession numbers MN639737–MN639763.

### Phylogenetic analysis of PUUV sequences

Phylogenetic trees were reconstructed with four novel and 18 published concatenated S, M, and L coding sequences or 202 partial S segment sequences of 365 nucleotides length. Published sequences of other hantaviruses were obtained from GenBank. Analysis was performed by Bayesian algorithms via MrBayes v.3.2.6 (https://sourceforge.net/projects/mrbayes/files/mrbayes/) on the CIPRES online portal [28]. A mixed nucleotide substitution matrix was specified in 4 independent runs of 10^7^ generations. Phylogenetic relations are shown as a maximum clade credibility phylogenetic tree with posterior probabilities for major nodes.

### Virus infection and titration

For immunofluorescence assay (IFA), VeroE6 and MGN-2-R cells were inoculated with 500 µl PUUV Osnabrück/V29 or PUUV Osnabrück/M43 supernatant in DMEM + 5% FCS as described previously [10]. Infected cells were fixed 10 days post infection with a 1:1 mixture of acetone and methanol for 20 min at − 20 °C. After fixation cells were dried, re-hydrated with phosphate-buffered saline (PBS) and incubated with nucleocapsid (N) protein-specific antibody 5E11 [13] diluted 1:1000 in PBS for 1 h at room temperature (RT). A secondary anti-mouse Alexa fluor 488 conjugated antibody (Abcam, Cambridge, UK) was used for detection of hantavirus proteins. Nuclei were stained with 4′,6-diamidino-2-phenylindole (DAPI, Thermo Fisher Scientific).

For titration studies of PUUV, MGN-2-R and VeroE6 cells were inoculated with 500 µl of the PUUV Osnabrück/V29 or PUUV Osnabrück/M43 virus isolate and passaged three times as described above. Supernatants of both cell lines were collected after passage three and frozen at − 80 °C. Subsequently, supernatants were serially diluted from 10^–1^ to 10^–7^ in DMEM containing 5% FCS in a 96-well plate with three replicates each. A volume of 100 µl of each dilution was added to 24 h old cell monolayers of VeroE6 cells in a 96-well plate. After incubation for 10 days, the virus titer was calculated using IFA for PUUV N protein detection as described above. Titers were calculated as 50% tissue culture infectious dose (TCID_50_)/ml by the Spearman/Kärber method [29] and mean titers of three experiments are given. Titers after isolation (passage 3 of original lung tissue-derived sample) were used for comparison.

### Generation of recombinant virus-like particles

For expression and generation of VLPs in HEK293 cells, a codon-optimized synthetic gene of the PUUV GPC of the strain Astrup [21] was purchased (GeneArt, Regensburg, Germany). The gene encoding the glycoproteins was PCR amplified using primer pair O GRS 101/O GRS 102 (aattaaGGTACCTCCAGAGGCGACACCCGGAACC and aattattAAGCTTTCAGGGCTTGTGTTCTTTGG) and the PCR product and the acceptor vector pHAN-1 (Roman-Sosa, unpublished) were digested with the restriction endonucleases KpnI and HindIII. The expression plasmid pHAN-2 was generated by standard molecular biology protocols. In this plasmid, the endogenous signal sequence of the PUUV Gn is substituted by the IgG-light chain signal sequence and a double strep-tag with a glycine/serine-rich linker between the tags. Then a permanently transfected HEK293 cell line was generated upon transfection of the cells and selection in the presence of geneticin at 0.5 mg/ml. The VLPs were affinity purified from the cell supernatants essentially as described [22].

### Generation of monoclonal antibodies against PUUV GPC

Recombinant VLPs were used for five immunizations of four weeks apart of female BALB/c mice. Hybridoma cells producing monoclonal antibodies (mAbs) were generated by standard fusion procedure [30, 31] and screened using a 2 µg/ml stock solution of VLPs according to an in-house ELISA protocol [32] and buffers without Tween. Resulting mAbs were analyzed by IFA and Western blot test for their reactivity to PUUV Osnabrück/V29, PUUV Sotkamo, PUUV Vranica and TULV Moravia.

### Immunofluorescence assay analysis of mAbs

VeroE6 cells were infected with PUUV Osnabrück/V29, PUUV Sotkamo, PUUV Vranica or TULV Moravia at multiplicity of infection (MOI) 0.1 in DMEM + 5% FCS. Infected cells were fixed 10 (PUUV Osnabrück/V29, Sotkamo) or 3 (PUUV Vranica, TULV Moravia) days post infection with a 1:1 mixture of acetone and methanol for 20 min at − 20 °C. After fixation cells were dried, re-hydrated with PBS and incubated with mAbs raised against GPC, 2E10 (diluted in PBS, 1:1), 5F12 (1:1), 3B12 (1:200), 5B8 (1:1), 5H1 (1:1), 4G10 (1:100), 1B12 (1:2), 1G9 (1:100), 8G4 (1:50), 1H7 (1:1), 2H11 (1:5), or N protein-specific antibody 5E11 (1:1000, [13]) for 1 h at RT. A secondary anti-mouse Alexa fluor 488 conjugated antibody (Abcam, Cambridge, UK) was used for detection of hantavirus proteins. Nuclei were stained with DAPI. After staining, slides were mounted on glass slides for imaging with Ibidi mounting medium (Ibidi, Gräfelfing, Germany).

### Western blot analysis of mAbs

VeroE6 cells were infected with PUUV Osnabrück/V29, PUUV Sotkamo, PUUV Vranica or TULV Moravia at MOI 0.1 in DMEM + 5% FCS. Cells were harvested 10 (PUUV Osnabrück/V29, Sotkamo) or 3 (PUUV Vranica, TULV Moravia) days post infection in SDS sample buffer (62.5 mM TrisHCl pH 6.8, 2% SDS,10% glycerol, 6 M Urea, 0.01% bromophenol blue, 0.01% phenol red) and proteins were separated by SDS PAGE, blotted onto polyvinylidenfluorid (PVDF) membranes. After blocking, the membranes were cut into strips and incubated over night with the antibodies 2E10 (1:1), 5F12 (1:1), 3B12 (1:200), 5B8 (1:1), 5H1 (1:1), 4G10 (1:100), 1B12 (1:2), 1G9 (1:100), 8G4 (1:50), 1H7 (1:1), 2H11 (1:5) or N protein-specific antibody 5E11 (1:1000, [13], all diluted in PBS-Tween 0.05%) at 4 °C. A horseradish peroxidase (HRP) labeled secondary goat anti-mouse IgG antibody diluted 1:3000 in PBS-Tween 0.05% (Bio-Rad, Hercules, CA, USA) was used for detection of hantaviral proteins. A rabbit anti-β-tubulin antibody (Abcam, Cambridge, UK) was used as a loading control.

### IgG ELISA analysis of chest cavity lavage of bank voles

Investigation of chest cavity lavage samples from bank voles was done by IgG ELISA using recombinant PUUV strain BaWa N protein, as described earlier [32]. The monoclonal antibody 5E11 was used as a positive control [13], chest cavity lavage of a IgG ELISA- and RT-PCR-negative bank vole was used as negative control. Chest cavity lavage samples with an optical density (OD) value below the lower cut-off value were considered as negative. Positive and doubtful samples were retested a second time. When the OD value of the ELISA was in a range between the lower and upper cut-off value defined according to our standard protocol [32], animals were considered doubtful. When the OD value was above the upper cut-off value, the samples were considered as positive.

## Results

### Isolation of PUUV from bank voles in the field

**Fig. 1:**
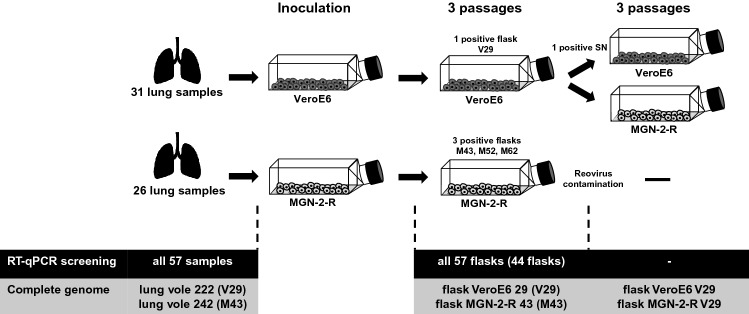
Schematic representation of the workflow. Bank voles were collected in forests within the district Osnabrück and a small piece of lung was taken by an incision in the chest area directly in the field. Lung tissue was meshed by grinding against a metal grid in a reaction tube containing 1 ml DMEM + 5% FCS and sterile filtered directly onto the cells. Cells were passaged three times until PUUV RT-qPCR screening. Supernatant of PUUV-positive flasks was taken and used for infection and further passaging in VeroE6 and MGN-2-R cells. Sequencing of complete genomes was done for PUUV RT-qPCR-positive passages and the corresponding original bank vole lung tissue. Isolates M52 and M62 were lost upon virus stock generation, presumably due to low viral load

Rodent trapping at five sites from April 11th to 12th, 2019 in the Osnabrück region resulted in the collection of 57 bank voles [25]. Dissection on site and inoculation of VeroE6 and bank vole MGN-2-R cells with homogenized lung samples resulted after three blind passages in four potential isolates that were detected by a novel PUUV RT-qPCR (Table S1, Fig. 1). Two of the potential candidates showed only low levels of PUUV RNA and were not able to consistently infect further passages (M52, M62). Quantification by RT-qPCR analysis of different tissues from these four bank voles confirmed lung tissue for most of the samples as having the highest PUUV RNA load, although it was detected in almost all other tissues investigated (Fig. S1).

### RT-PCR and IgG ELISA analysis of bank voles

RT-qPCR investigation of lung tissues of all 57 bank voles resulted in the detection of hantavirus RNA in 44 animals (Tables 1, S1, [25]). PUUV RNA-positive animals originated from all five trapping sites. Serological analysis of chest cavity lavages detected PUUV N protein reactive antibodies in 24 of 57 bank voles (Tables 1, S1). Five additional animals, positive for PUUV RNA, were found to be equivocal in our serological test. All 24 antibody-positive animals were also found to be PUUV RNA positive, indicating a high number of persistently infected voles. Fifteen additional bank voles were only positive for PUUV RNA, but not for anti-PUUV antibodies, indicating a high number of acutely infected animals in spring in this region (Table 1). Interestingly three of the four potential isolates originated from seronegative bank voles (Table S1).

**Table 1:** Results of molecular and serological *Puumala orthohantavirus* testing of trapped bank voles

Trapping site	Total number of bank voles	RT-qPCR positive^a^/total number investigated	IgG ELISA doubtful/positive/total number investigated	RT-qPCR positive and IgG ELISA doubtful/positive/total number investigated	Only RT-qPCR positive^b^/total number investigated
Schledehausen Forest	18	16/18	3/9/18	3/9/18	4/18
Schledehausen Field	21	16/21	1/9/21	1/9/21	6/21
Ellerbeck	5	5/5	1/2/5	1/2/5	2/5
Astrup I	6	4/6	0/3/6	0/3/6	1/6
Astrup II	7	3/7	0/1/7	0/1/7	2/7
Total	57	44/57	5/24/57	5/24/57	15/57

*RT-qPCR* real-time reverse transcription-polymerase chain reaction targeting the *Puumala orthohantavirus* S segment

^a^Results of RT-qPCR-positive animals were partly already published in [25]

^b^RT-qPCR positive, but IgG ELISA negative

### Characterization of the PUUV isolates

**Fig. 2:**
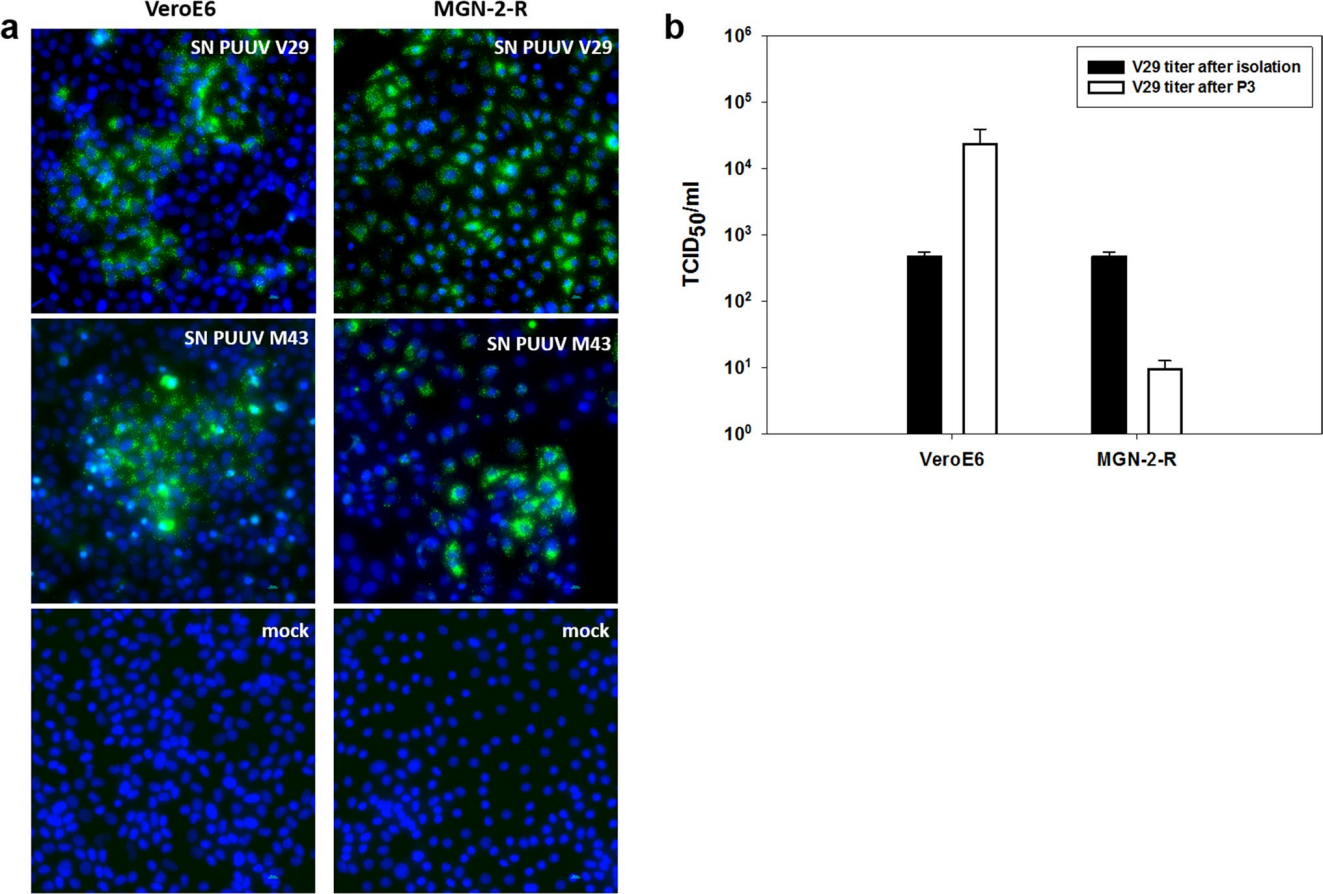
Infection studies of PUUV isolates in VeroE6 and MGN-2-R cells. **a** Immunofluorescence analysis of VeroE6 and MGN-2-R cells inoculated with supernatants (SN) of PUUV Osnabrück/V29, isolated on VeroE6 cells, or PUUV Osnabrück/M43, isolated on MGN-2-R cells. PUUV-inoculated and mock-infected cells were fixed 10 days post infection and stained with nucleocapsid protein-specific antibody 5E11 and a secondary anti-mouse Alexa fluor 488 conjugated antibody. Nuclei were stained with DAPI. **b** Determination of virus titers of PUUV Osnabrück/V29 isolate (TCID_50_/ml) after three passages (P3) in VeroE6 and MGN-2-R cells (white columns) in comparison to titers directly after isolation in VeroE6 cells (black columns). Titers were obtained by immunofluorescence staining of 96-well plates 10 days post inoculation

**Fig. 3:**
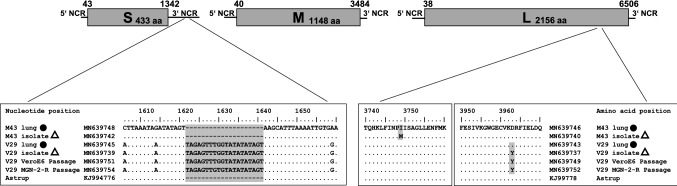
Complete genome analysis of PUUV isolates. Complete PUUV genomes isolated from lung tissues and positive cell line passages were determined by HTS and dideoxy-chain termination sequencing in combination with RNA ligation to obtain complete NCRs. Nucleotide sequence insertion in the S segment NCR and amino acid exchanges in the L segment encoded RdRP are compared. Black dots indicate sequences derived from lung tissue and triangles indicate sequences obtained from cell culture passages. The complete genome of PUUV strain Astrup was used as a reference sequence ([21]; GenBank accession numbers: KJ994776-78). Coding regions of the three segments are indicated by numbers. NCR, non-coding region; M43, PUUV Osnabrück/MGN-2-R 43; V29, PUUV Osnabrück/VeroE6 29

Two isolates (Osnabrück V29 and Osnabrück M43) were obtained by passaging in VeroE6 or MGN-2-R cells, which reached titers of almost 10^3^ TCID_50_/ml (Fig. 2a and b, titer after isolation). Shot-gun and hybrid-capture-mediated HTS of both isolates resulted in the generation of complete genome sequences which are identical in sequence to the respective original strain in bank vole lung tissue except for one amino acid (aa) exchange each in the RNA-dependent RNA polymerase (RdRP) of strain M43 (I3749M) and V29 (D3963Y; Fig. 3).

**Fig. 4:**
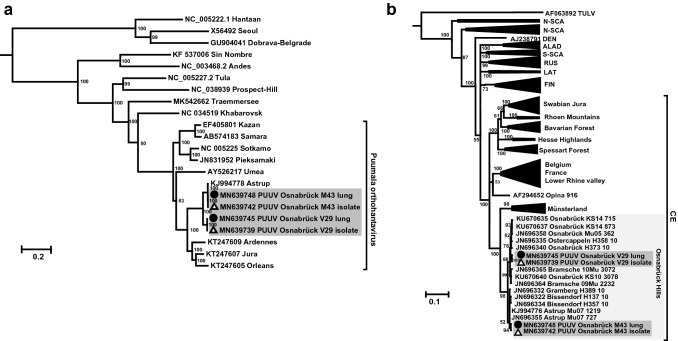
Hantavirus phylogenetic trees. Hantavirus phylogenetic tree of concatenated S, M, and L coding sequences of 18 published and four novel complete genomes (**a**). Partial S segment coding sequences of 365 nucleotides length, reconstructed with four novel and 202 published partial sequences (**b**). New PUUV isolates (GenBank accession numbers: MN639737-MN639742) are indicated with triangles and sequences derived from their original lung tissue (GenBank accession numbers: MN639743-MN639748) are labeled with black dots. Published sequences of other hantaviruses are labeled with GenBank accession numbers. Novel sequences are highlighted in gray. Posterior probabilities for major nodes of the maximum clade credibility phylogenetic tree are displayed. Analysis was performed by Bayesian algorithms via MrBayes v.3.2.6 (https://sourceforge.net/projects/mrbayes/files/mrbayes/) on the CIPRES online portal [28]. A mixed nucleotide substitution matrix was specified in 4 independent runs of 10^7^ generations. Scale bar indicates nucleotide substitutions per site. For clarity, previously characterized PUUV clades from other parts of Europe are shown in simplified form. CE, Central European; LAT, Latvian; ALAD, Alpe-Adrian; S-SCA, South Scandinavian; N-SCA, North Scandinavian; RUS, Russian; FIN, Finnish; DEN, Danish

The genome organization of the novel PUUV isolates indicated the typical sequence elements for PUUV: The small (S) segment encodes an N protein of 433 aa residues and a putative NSs protein of 90 aa in an + 1 overlapping reading frame, the medium (M) segment codes for the 1148 aa GPC and the large (L) segment for the RdRP of 2156 aa (see Fig. 3, GenBank accession numbers: MN639737–MN639748). Phylogenetic analysis of the concatenated S, M and L segment coding sequences grouped the novel isolates together with Astrup prototype strain in sister relationship to PUUV sequences from France (Fig. 4a). The phylogenetic analysis of a partial S segment sequence of the novel isolates and representative strains of all PUUV clades and subclades from Germany confirmed the close relationship of the new isolates to the Osnabrück hills subclade (Fig. 4b).

The PUUV Osnabrück M43 isolate was found to be contaminated by a bank vole reovirus; HTS derived sequences of the passaged reovirus (GenBank accession numbers: MN639755–MN639763) showed a strong similarity to a bank vole reovirus strain, but much lower similarity to a common vole reovirus [33]).

The non-reovirus contaminated isolate Osnabrück V29 from VeroE6 cells was found to have an insertion of 20 nucleotides in the 3′ non-coding region (NCR) when compared to the other isolate and the Astrup reference sequence (Fig. 3). However, this insertion was also found in the original lung sample and therefore no cell culture-specific adaptations were observed in the NCRs of both virus isolates (Fig. 3).

### Passaging experiment for isolate V29 in vole and VeroE6 cells

V29 isolate was passaged in parallel again in VeroE6 cells and in MGN-2-R cells (Figs. 1 and 2). This passaging resulted in no further mutations (GenBank accession numbers: MN639749–MN639754). However, the virus isolate passaged in VeroE6 cells is accompanied by an increase in the virus titer to 10^4^ TCID_50_/ml (Fig. 2). In contrast, the passaging of the Osnabrück V29 strain in MGN-2-R cells resulted in a decreased virus titer. As no cytopathic effect was observed, virus detection for titration in both cell lines was done by immunofluorescence assay using an N protein-specific monoclonal antibody (Fig. 2a).

### Development of monoclonal antibodies against the new PUUV isolate

**Fig. 5:**
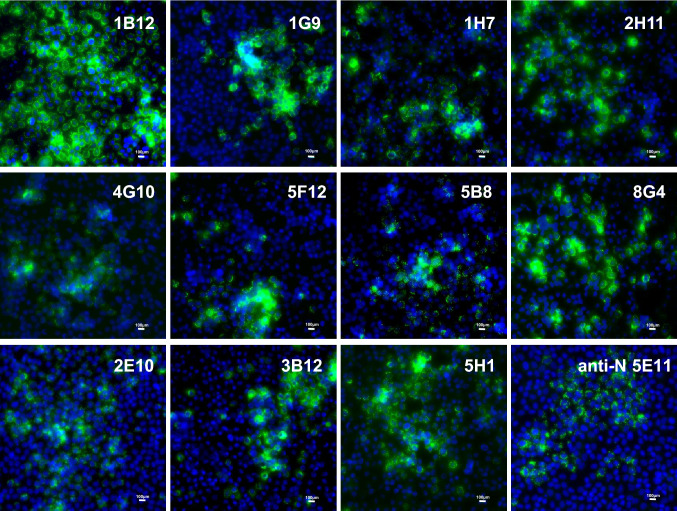
Reactivity of novel PUUV GPC-specific monoclonal antibodies with hantavirus-infected VeroE6 cells in immunofluorescence assay (IFA). Antibodies were generated by immunization of BALB/c mice with GPC-derived virus-like particles of PUUV strain Astrup. After screening and subcloning, monoclonal antibodies were tested in IFA. VeroE6 cells were infected with PUUV Osnabrück V29 isolate on coverslips and fixed for IFA after 10 days. The monoclonal antibodies were administered for 1 h at RT. Detection of the specific antibody binding was done using an anti-mouse Alexa fluor 488 conjugated antibody. After staining, coverslips were mounted on glass slides for imaging

Eleven monoclonal antibodies were produced in this study by immunization of mice with PUUV strain Astrup GPC-derived VLPs. Evaluation of the virus isolate Osnabrück V29 using these monoclonal antibodies resulted in typical immunofluorescence patterns in the cytoplasm (Fig. 5). Further analysis by Western blot test using a lysate of isolate Osnabrück V29 from VeroE6 cells suggested that the majority of anti-GPC antibodies are directed against conformational epitopes; however, some recognize linear epitopes in Gc or Gn (Table 2). Subsequent evaluation of the reactivity of these monoclonal antibodies with other PUUV strains and TULV strain Moravia indicated some level of cross-reactivity for some of them (Table 2).

**Table 2:** Reactivity of monoclonal antibodies with hantavirus-infected VeroE6 cells in immunofluorescence assay (IFA) and Western blot test (WB)

Antibody	PUUV V29 Osnabrück		PUUV Sotkamo		PUUV Vranica		TULV Moravia	
	IFA	WB	IFA	WB	IFA	WB	IFA	WB
1B12	++	−	−	−	−	−	−	−
5H1	++	−	−	−	−	−	−	−
2H11	++	−	−	−	−	−	−	−
1G9	++	−	(+)	−	+	−	−	−
5F12	++	−	+	−	+	−	−	−
3B12	++	−	+	−	++	−	++	−
8G4	++	−	+	−	++	−	+	−
4G10	++	−	+	−	(+)	−	++	−
1H7	++	+(Gn)	−	+	−	+	−	−
5B8	++	+(Gc)	+	+	+	−	+	−
2E10	(+)	+(Gc)	(+)	+	++	−	+	−

VeroE6 cells were inoculated with Puumala virus (PUUV) Osnabrück/V29, PUUV Sotkamo, PUUV Vranica or Tula virus (TULV) strain Moravia. Infected cells were fixed 10 (PUUV Osnabrück/V29, Sotkamo) or 3 (PUUV Vranica, TULV Moravia) days post infection for immunofluorescence assays or collected in sample buffer for Western blot analysis. After fixation or Western blot transfer, novel GPC-specific mAbs 2E10, 5F12, 3B12, 5B8, 5H1, 4G10, 1B12, 1G9, 8G4, 1H7, and 2H11 were administered. Gn- and Gc-reactive mAbs were assigned where possible according to molecular weight of the immunoreactive bands in Western blot analysis

− negative; (+) weak reactivity; + positive; ++ strongly positive

## Discussion

Here, we describe the first isolation of a Central European PUUV strain. This strain of the Central European lineage increases the available panel of PUUV isolates: Currently available isolates Sotkamo, Umea, Vranica, and Kazan, belong to the clades Finnish, North Scandinavian, most likely North Scandinavian, and Russian, respectively [34]. The PUUV-like Hokkaido virus strain Kitahiyama128 originates from Japan [12]. In our study, the isolation was based on an in-field dissection and inoculation of cells to prevent freeze/thaw cycles. The subsequent investigation of all 57 bank voles indicated that three of four isolates originated from anti-PUUV-seronegative voles. This finding illustrates that a serological test in the field might be misleading in selection of samples for successful virus isolation. Instead, an on-site molecular assay may enhance the chance for a successful virus isolation. Nevertheless, the approach used here still indicates the challenges of hantavirus isolation; only four isolates were obtained from a total of 15 acutely infected bank voles. In addition, the determination of the complete genome sequences of two isolates including the NCRs expands our knowledge on the sequence diversity of PUUV strains within the different regions of the genome. Moreover, the hybrid-capture-based enrichment of PUUV sequences allows a rapid determination of the complete genome and underlines the value of this workflow for hantavirus surveillance and molecular evolution studies [35]. A phylogenetic analysis of partial S segment nucleotide sequences confirmed the previously reported subclades of PUUV in Germany; the novel isolates belong to the subclade Osnabrück hills within the Central European clade. The position within the phylogenetic tree also confirms the local evolution pattern of PUUV reported before [23, 36].

The observed high level of RT-qPCR-positive bank voles (44/57; 77%) confirms the district of Osnabrück in spring 2019 as a hantavirus outbreak region [25]. The PUUV RNA detection rate was similarly high at all five trapping sites of bank voles. Although 2019 was identified as a hantavirus outbreak year in Germany, the distribution of notified human PUUV cases was not as homogeneous as in previous outbreak years [25].

The passage of the PUUV strains for isolation resulted in non-synonymous nucleotide exchanges in the L segment responsible for single amino acid exchanges in the RdRP (I3749M in M43 and D3963Y in V29). The substituted amino acid residues are each very similar in their properties and, presumably, might not influence protein function. A more divergent adaptation at position S2053F has previously been observed for PUUV strain Kazan [8, 37]. Although in this previous study nucleotide exchanges in the NCR of the S segment were observed [37], here we did not find relevant mutations in this region after passaging in cell culture. The V29 strain showed an insertion in the 3′ NCR, but this insert was also found in the original lung material used for isolation. Additionally, this sequence insert was found in another sequence from the same region (JN696358.1, [36]).

The isolate V29 was shown to replicate in VeroE6 and a bank vole kidney cell line. The low titer in the bank vole MGN-2-R cell line might be due to the evolutionary lineage origin of this cell line (Carpathian lineage); in Central Europe PUUV is harbored by the Western evolutionary lineage with spillover to the Carpathian lineage in regions with sympatric occurrence of both [24]. In line with the assumption of an association of a PUUV clade with an evolutionary bank vole lineage, the Vranica PUUV strain replicated in MGN-2-R cells, but not in bank vole kidney cells of another evolutionary lineage [9, 10]. Interestingly, replication of PUUV-like Hokkaido virus in cells of its host, the gray red-backed vole, was comparable to PUUV infection [12]. Future investigations in cell lines and animals of different bank vole lineages are required to confirm this conclusion directly.

The orthoreovirus contamination of one of the PUUV isolates illustrates that bank voles may harbor additional infectious agents that may influence the susceptibility to PUUV infections or their outcome. Of note, in bank voles several viruses have been detected, i.e., polyoma-, herpes- and hepaciviruses [38–41], but also bacterial agents and endoparasites [42–44]. Similarly, a hantavirus isolation approach was previously hampered by the coinfection by a striped field mouse adenovirus [45]. Future investigations are needed to evaluate potential influences of coinfections in bank voles.

It has been shown that hantavirus Gn and Gc form complex spike-shaped structures [46] that build conformational epitopes [17, 18]. Therefore, we selected an immunization procedure using PUUV-GPC-derived VLPs, as the organization of the glycoproteins resembles the one of the virion. A panel of eleven monoclonal antibodies was produced here and all of them were reactive with the new PUUV isolate in immunofluorescence assay. The staining pattern, which is reminiscent of the one of the secretory pathway organelles, i.e., the Golgi apparatus and the endoplasmic reticulum, suggests that the epitopes recognized by these antibodies are already accessible during the maturation process of the proteins. Interestingly, some of the monoclonal antibodies recognize linear epitopes as revealed by a Western blot assay. Although preliminary results suggest that the antibodies do not neutralize the virus when tested individually, synergistic effects with a protective effect cannot be ruled out yet as shown for anti-Ebola virus monoclonal antibodies [47]. Therefore, the novel antibodies represent a useful tool for further experimental, diagnostic, and therapeutic applications.

In conclusion, the PUUV isolate described here replicates in a bank vole cell line and its N and GPC proteins can be detected by specific monoclonal antibodies. Therefore, this isolate will be useful for further studies on the virulence markers of Central European PUUV, its reservoir host association and the route of pathogenicity in the bank vole model. The novel GPC-specific monoclonal antibodies will enable future studies on virus entry and important domains for exposed immunogenic regions.

## Electronic supplementary material

Below is the link to the electronic supplementary material.

Supplementary file1 (DOCX 33 kb)
