# Supplementary material for: Isolation and characterization of new Puumala orthohantavirus strains from Germany
Source: Virus Genes. 2020 Apr 23;56(4):448–60. doi: 10.1007/s11262-020-01755-3 (PMC7329759; doi:10.1007/s11262-020-01755-3)
Supplement: Supplementary file 1 — Supplementary file1 (DOCX 33 kb) [file 11262_2020_1755_MOESM1_ESM.docx]

**Supplementary Material**

**Supplementary Figure S1:**  **Viral RNA load analysis of bank vole tissue.** For Puumala virus (PUUV) nucleic acid detection, RNA was extracted from homogenized heart, lung, trachea, liver, spleen, kidney, and brain tissue using QIAzol Lysis Reagent followed by specific quantitative real-time RT-PCR. For quantification of the number of RNA copies/µl and sample, the PUUV S segment sequence of nucleotides 83-355 was *in vitro* transcribed from a plasmid. The transcribed RNA was serially diluted from 10^-2^ to 10^-11^ ng/ml with 700 RNA copies/µl limit of detection (LOD). Viral load as RNA copies/µl was determined in triplicates for organs of the four isolation positive animals.

**Supplementary Table S1: Results of the investigations of all bank voles trapped for PUUV isolation (isolates are indicated in bold face).**

| **Cell culture number** | **Trapping number LKOS** | **Trapping date** | **Trapping site** | **RT-qPCR** | **lung sample Ct-value** | **Results of IgG ELISA*** | **First RT-qPCR positive passage** | **Cells used for isolation** |
| --- | --- | --- | --- | --- | --- | --- | --- | --- |
| V1 | 164 | 11.04.2019 | Schledehausen Forest | positive | 20.05 | negative |  | VeroE6 |
| V2 | 165 | 11.04.2019 | Schledehausen Forest | positive | 19.69 | doubtful |  | VeroE6 |
| V3 | 166 | 11.04.2019 | Schledehausen Forest | positive | 25.51 | negative |  | VeroE6 |
| V4 | 167 | 11.04.2019 | Schledehausen Forest | positive | 21.28 | positive |  | VeroE6 |
| V5 | 168 | 11.04.2019 | Schledehausen Forest | positive | 20.07 | positive |  | VeroE6 |
| V6 | 169 | 11.04.2019 | Schledehausen Forest | positive | 22.77 | positive |  | VeroE6 |
| V7 | 170 | 11.04.2019 | Schledehausen Forest | positive | 20.95 | positive |  | VeroE6 |
| V8 | 171 | 11.04.2019 | Schledehausen Forest | negative | - | negative |  | VeroE6 |
| V9 | 172 | 11.04.2019 | Schledehausen Forest | positive | 21.21 | positive |  | VeroE6 |
| V10 | 173 | 11.04.2019 | Schledehausen Forest | positive | 19.71 | positive |  | VeroE6 |
| V11 | 174 | 11.04.2019 | Schledehausen Forest | positive | 18.55 | doubtful |  | VeroE6 |
| V12 | 175 | 11.04.2019 | Schledehausen Forest | positive | 18.00 | doubtful |  | VeroE6 |
| V13 | 190 | 11.04.2019 | Schledehausen Field | positive | 22.24 | negative |  | VeroE6 |
| V14 | 191 | 11.04.2019 | Schledehausen Field | negative | - | negative |  | VeroE6 |
| V15 | 192 | 11.04.2019 | Schledehausen Field | negative | - | negative |  | VeroE6 |
| V16 | 193 | 11.04.2019 | Schledehausen Field | positive | 21.56 | negative |  | VeroE6 |
| V17 | 194 | 11.04.2019 | Schledehausen Field | negative | - | negative |  | VeroE6 |
| V18 | 195 | 11.04.2019 | Schledehausen Field | positive | 24.32 | positive |  | VeroE6 |
| V19 | 196 | 11.04.2019 | Schledehausen Field | negative | - | negative |  | VeroE6 |
| V20 | 206 | 11.04.2019 | Ellerbeck | positive | 26.77 | negative |  | VeroE6 |
| V21 | 207 | 11.04.2019 | Ellerbeck | positive | 22.53 | positive |  | VeroE6 |
| V22 | 211 | 11.04.2019 | Astrup I | negative | - | negative |  | VeroE6 |
| V23 | 212 | 11.04.2019 | Astrup I | negative | - | negative |  | VeroE6 |
| V24 | 213 | 11.04.2019 | Astrup I | positive | 23.20 | negative |  | VeroE6 |
| V25 | 214 | 11.04.2019 | Astrup I | positive | 22.41 | positive |  | VeroE6 |
| V26 | 215 | 11.04.2019 | Astrup I | positive | 18.92 | positive |  | VeroE6 |
| V27 | 220 | 11.04.2019 | Astrup II | positive | 16.94 | negative |  | VeroE6 |
| V28 | 221 | 11.04.2019 | Astrup II | positive | 21.97 | positive |  | VeroE6 |
| **V29** | **222** | **11.04.2019** | **Astrup II** | **positive** | **17.22** | **negative** | **2** | **VeroE6** |
| V30 | 223 | 11.04.2019 | Astrup II | negative | - | negative |  | VeroE6 |
| V31 | 224 | 11.04.2019 | Astrup II | negative | - | negative |  | VeroE6 |
| M41 | 240 | 12.04.2019 | Schledehausen Forest | negative | - | negative |  | MGN-2-R |
| M42 | 241 | 12.04.2019 | Schledehausen Forest | positive | 22.20 | positive |  | MGN-2-R |
| **M43** | **242** | **12.04.2019** | **Schledehausen Forest** | **positive** | **22.76** | **negative** | **3** | **MGN-2-R** |
| M44 | 243 | 12.04.2019 | Schledehausen Forest | positive | 17.85 | positive |  | MGN-2-R |
| M45 | 244 | 12.04.2019 | Schledehausen Forest | positive | 19.10 | negative |  | MGN-2-R |
| M46 | 245 | 12.04.2019 | Schledehausen Forest | positive | 18.77 | positive |  | MGN-2-R |
| M47 | 263 | 12.04.2019 | Schledehausen Field | positive | 17.03 | doubtful |  | MGN-2-R |
| M48 | 264 | 12.04.2019 | Schledehausen Field | positive | 16.74 | positive |  | MGN-2-R |
| M49 | 265 | 12.04.2019 | Schledehausen Field | negative | - | negative |  | MGN-2-R |
| M50 | 266 | 12.04.2019 | Schledehausen Field | positive | 18.98 | positive |  | MGN-2-R |
| M51 | 267 | 12.04.2019 | Schledehausen Field | positive | 22.49 | negative |  | MGN-2-R |
| **M52** | **268** | **12.04.2019** | **Schledehausen Field** | **positive** | **23.52** | **positive** | **3*** | **MGN-2-R** |
| M53 | 269 | 12.04.2019 | Schledehausen Field | positive | 18.31 | negative |  | MGN-2-R |
| M54 | 270 | 12.04.2019 | Schledehausen Field | positive | 23.04 | positive |  | MGN-2-R |
| M55 | 271 | 12.04.2019 | Schledehausen Field | positive | 19.29 | positive |  | MGN-2-R |
| M56 | 272 | 12.04.2019 | Schledehausen Field | positive | 20.09 | negative |  | MGN-2-R |
| M57 | 273 | 12.04.2019 | Schledehausen Field | positive | 18.99 | positive |  | MGN-2-R |
| M58 | 274 | 12.04.2019 | Schledehausen Field | positive | 26.58 | negative |  | MGN-2-R |
| M59 | 275 | 12.04.2019 | Schledehausen Field | positive | 19.35 | positive |  | MGN-2-R |
| M60 | 276 | 12.04.2019 | Schledehausen Field | positive | 20.43 | positive |  | MGN-2-R |
| M61 | 286 | 12.04.2019 | Ellerbeck | positive | 18.81 | doubtful |  | MGN-2-R |
| **M62** | **287** | **12.04.2019** | **Ellerbeck** | **positive** | **19.21** | **negative** | **3*** | **MGN-2-R** |
| M63 | 288 | 12.04.2019 | Ellerbeck | positive | 19.72 | positive |  | MGN-2-R |
| M64 | 290 | 12.04.2019 | Astrup I | positive | 27.58 | positive |  | MGN-2-R |
| M65 | 299 | 12.04.2019 | Astrup II | negative | - | negative |  | MGN-2-R |
| M66 | 300 | 12.04.2019 | Astrup II | negative | - | negative |  | MGN-2-R |

Ct, cycle threshold; LKOS, district Osnabrück.

*negative, optical density (OD) < lower cut-off value; positive, OD > upper cut-off value; doubtful: lower cut-off value < OD < upper cut-off value

* PUUV isolates were lost during generation of virus stocks after third passage.
